# University Entrance Examinations in Spain: Using the Construct Comparability Approach to Analyze Standards Quality

**DOI:** 10.3389/fpsyg.2020.00127

**Published:** 2020-02-04

**Authors:** Alejandro Veas, Leandro Navas, Teresa Pozo-Rico, Pablo Miñano

**Affiliations:** Department of Developmental Psychology and Didactics, University of Alicante, Alicante, Spain

**Keywords:** University Entrance Examinations, academic achievement, construct comparability approach, rasch partial credit model, higher education

## Abstract

In recent years, important methodological attempts have been made to explore the comparability of examination standards, especially in the context of certifications and university entrance. The present study aimed to explore the use of a construct comparability approach through a comparative analysis of the academic scores on 15 subjects from Spanish University Entrance Examinations in the Valencian Community, with a sample of 22,996 students in the call of June 2018. We employed the Rasch partial credit model as an estimation method, counting each subject as the item of an instrument related to academic achievement. The results confirmed the unidimensionality assumption and the goodness of fit of the model in relation to all subjects, although no discrimination between high and low ability students was detected because of the lack of monotonicity of the score categories. We observed that the level of difficulty of the subjects was appropriate to the students’ ability levels. Important conclusions have been drawn for the improvement of the standard qualification process, and future research directions have been proposed.

## Introduction

Across different countries, standard examinations constitute a formal procedure to select high school students based on academic achievement in different courses. This type of procedure has served as a governance instrument to provide consistent required standards of achievement, objective examination conditions, and grading procedures (Neumann et al., 2011).

The use of improved measures of academic achievement can be considered a positive consequence of the desire to increase economic growth and competitiveness (Sahlberg, 2006). Moreover, there has been a notable research interest in understanding how students’ achievement can be improved with analysis of the cognitive, motivational, and contextual variables involved in causal or predictive models (Valle et al., 2008; Dicke et al., 2018). For these reasons, it is also relevant to study how different types of examinations (tests or written exams) use the required psychometric properties according to specific goals determined by educational administrations (Raykov and Pohl, 2013). This article aims to explore the measurement quality of the 2018 University Entrance Examinations in the Spanish territory of the Valencian Community, based on the construct comparability approach (Coe, 2008).

### The University Entrance Examinations in Spain

In Spain, the University Entrance Examinations (known as *PAU*) are formal procedures for access to higher education, undertaken by those who have previously obtained the Spanish Baccalaureate certificate (*Bachillerato*); these are based on examination standards of mandatory and modality subjects that have been studied during the previous course. Depending on the subjects, examinations have different formats, such as essays (e.g., History of Spain and Spanish Language), analyzing visual images (Art History), texts on a specific topic of large or short extension (e.g., English Language and Latin Language), or solving problems (e.g., Economy), among others. Moreover, it must be noted that there is no unique examination for the whole country; rather, each community is autonomous and has the objective to design specific examinations for the students living within that community. The mean grade obtained from these examinations is weighted with the Baccalaureate grade, and a final evaluation is obtained to access the chosen undergraduate degree that may be located in any part of the country.

As the Spanish University Entrance Examinations are crucial for the future of thousands of students every year, it is necessary to consider the role of research assessment in the field of education. Within this context, the analysis of the process and the results obtained, as well as the employment of the distinct procedures, are relevant to ensure equality and equity of opportunities in higher education access.

In quantitative research, statistical methods have been applied to investigate the necessary conditions for measuring academic achievement objectively, with the correct design and use of measurement instruments – for example, value added models and multilevel models from a longitudinal perspective (Blanco et al., 2009; López-Martín et al., 2014).

With respect to the PAU, important research was conducted by Gaviria (2005), where different statistical techniques were applied – classic, ordinary least squares, multilevel, and mean and standard deviation equality methods – to match the grade obtained in the Baccalaureate with that obtained in the PAU; the last served as anchor, as the examination was the same for all participants. The results showed that the non-classical method produced worse results than classical methods, improving justice in student selection. Apart from this study, no other relevant research is found beyond quantitative analyses, which refer to group differences in a specific context (Ruiz et al., 2013). For this reason, this study aimed to fulfill the existing limitations by adding new comparability analyses of standard examinations.

### Advances and Limitations of Standard Examinations Comparability

Traditionally, standardized achievement tests are considered the most objective procedure, as they reflect a unidimensional construct that is highly dependent upon students’ cognitive abilities (Hübner et al., 2019). Different international organizations have clarified the improvements in the design and implementation of international standardized tests such as TIMMS (*Trends in International Mathematics and Science Study*), PIRLS (*Progress in International Reading Literacy Study*), IALS (*International Assessment of Literacy Survey*), and especially PISA (*Programme for International Student Assessment*).

On the other hand, written examinations based on grades are considered a multidimensional construct, in which teachers use different criteria (Guskey, 2006). In this area, multiple studies have claimed the impact of various frames of reference. For example, Westphal et al. (2016) found that teachers’ judgments were associated with the socioeconomic composition of the classroom in a sample of 3,285 math fourth graders. Zimmermann et al. (2013) showed, in a longitudinal study of 1,045 students from Grade 5 to Grade 9, that external problems are reflected in teacher-given grades more than in standardized achievement tests.

Given the possible factors associated with grading, there have been several attempts to improve objective grading criteria in Europe, as written examinations are crucial in educational systems, especially for obtaining institutional certificates, or selecting students for higher education (Newton, 2005, 2010). For instance, the implementation of the central Abitur examination in Germany is remarkable (Kuehn, 2012). Although these examinations present differences in procedures or subjects between each German state, their higher level of standardization means that these grades are less affected by factors related to the schools or the teachers (Neumann et al., 2011). On the other hand, important methodological advances have been implemented in England for examinations used in academic qualifications such as the General Certificate of Secondary Education (GCSE, taken by students aged 16) and General Certificate of Education Advanced level (GCE A level, taken by students aged 18) (Coe, 2008; Newton et al., 2007). In this context, special attention has been given to inter-subject comparability using a variety of statistical procedures, including pair analyses, common examinee linear models, and item response theory models (Coe et al., 2008).

Inter-subject comparability of examination standards constitutes an educational need to apply statistical aligns when grades from different subjects are used for specific objectives. When this is possible, academic achievement can be measured as the level of an individual’s skill in a specific examination of a certain difficulty. In the context of comparing academic grades, it is also important to notice that we can only compare those measuring a shared construct. For this reason, the concept *construct comparability approach* constitutes a formal theoretical framework in which statistical applications are applied (Coe, 2010).

### Use of the Rasch Model Within the Construct Comparability Approach

Different authors have developed advanced psychometric analyses for the comparison of subject examinations. In this context, more specifically, the Rasch measurement model was chosen as the most appropriate, given the theoretical framework and the complexity of data. Coe (2008) implemented it in a sample of nearly 6,000 candidates who took GCSEs in 2004, including the exploration of Differential Item Functioning (DIF). Recently, He et al. (2018) also applied the Rasch analysis to both the GCSE and GCE A levels over a period of 4 years, in order to establish the consistency of difficulty parameters and grade comparison in the same country. Other countries, such as Tasmania, have approved educational policies based on the formal application of the Rasch model to the alignment of statistical standards (Tasmanian Qualification Authority [TQA], 2006, 2007).

The Rasch (1980) model is regarded as the most renowned of IRT models, providing a method based on the calibration of ordinal data from a shared measurement scale and enabling one to test conditions such as dimensionality, linearity, and monotonicity. This model analyses the difficulty of items and individuals’ ability on the same scale, employing a logarithmic function to test the probability of a subject to correctly respond to an item. Use of the same measurement scale established homogeneous intervals, meaning that the same difference between the difficulty parameter of an item and the ability of a subject involves equal probability of success along the entire scale (Preece, 2002).

According to comparability criteria, we started by considering each of the courses as a specific item, with a range of grades from 1 to 10, which implies various degrees or categories of success. In this case, the partial credit model (PCM) (Wright and Masters, 1982) enabled the analysis of the difficulty in achieving a specific score for each of the subjects separately, following the Rasch methodology. In this study, the use of PCM is justified in the fact that, in Spain, the same grades obtained in different examinations are not necessarily related to the same level of effort (He et al., 2018).

The formula of the model is as follows:

$$\text{Ln}\,\left(P_{n\,i\,j}/P_{n\,i\,\left(j-1\right)}\right)=B_{n}-D_{i}\,F_{i\,j}=B_{n}-D_{i\,j}$$

where:

*P _*nij*_* is the probability of subject *n* responding correctly to item *i* observed in category *j*;

*B _*n*_* is the measured ability of subject *n*;

*D _*i*_* is the measured difficulty of item *i*; and

*F _*ij*_* is the calibration measured for item *i* in category *j* compared to category *j-1*, the point at which categories *j-1* and *j* are equally likely compared to the measurement of the item (Masters, 1982).

### The Present Study

The use of the Rasch model for analyzing inter-subject comparability has been employed in different countries (Coe, 2008; Korobko et al., 2008). Based on the literature review, the present study aimed to apply the Rasch PCM in the Spanish University Entrance Examinations taken in the Valencian Community, according to the construct comparability approach, which was developed in England over the last decades. Concretely, three main objectives were followed, specifically (1) to analyze the unidimensionality of the measures; (2) to compare the fit statistics and difficulty parameters between the different subjects, and (3) to compare the distribution of difficulty level of the subject grades along the latent trait. Given that no previous IRT analysis has been conducted on these examinations in Spain, there are no directional hypotheses to be determined.

## Materials and Methods

### Sample

The sample was taken from all students in the Valencian Community that participated in the Spanish University Entrance Examinations in the last call of June 2018. The community is located on the east coast of the country and comprises three provinces: Alicante, Valencia, and Castellón. A total of 22,996 students were considered: 10,015 students took the exam in the province of Alicante (43.55% of the total sample), 2248 students in the province of Castellón (9.77% of the total sample), and 10,733 students in the province of Valencia (46.77%). For each province, examinations were taken in different public universities or venues belonging to these universities (extension areas where specific degrees are taught). Approximately 60% were females.

### Measures

The Spanish University Entrance Examinations from the call of June 2018 were considered for further analysis. A total of 24 subjects (described in the section “Results”) were first considered, accounting for both mandatory and modality subjects. All the examinations have correction standards previously approved by the qualification board. In this sense, corrections criteria are defined and given top scores for each specific question in each exam, together with a qualitative instruction that helps examiners ensure objectivity. For all the examinations, the lowest score is 0 and the highest is 10, with the sum of the grade obtained in each question based on raters’ assignments. These qualification criteria are public and available on the website of the Valencian Community Government (2019).

### Procedure

Necessary permission was first obtained by the University Regulation Service, an institution belonging to the Valencian Community Government; it provided the grades from all students enrolled in the University Entrance Examinations in the three provinces of the Valencian Community – Alicante, Valencia, and Castellón – at the call of June and July 2018. For the present research, data from June 2018 were taken for the analysis. This study was approved by the Institutional Review Board and complied with the Ethical standards of the 1964 Helsinki Declaration and its later amendments, or comparable ethical standards.

### Data Analysis

For the present study, the construct comparability approach was applied based on the assumption that it is possible to compare the qualifications obtained by the students for the subjects involved in the higher education selection process. The software Winsteps version 4.4.0 (Linacre, 2019) was employed to implement Rasch PCM, where a joint maximum likelihood estimation was realized. In this model, each of the included subjects was considered an item of the same instrument that contributes to the measurement of the construct academic achievement.

First, and according to the Rasch assumptions, unidimensionality was tested with a principal component analysis of residuals. According to Linacre (1998, 2002), the eigenvalues obtained for each contrast comparison should be no more than 2. Moreover, the estimation process of the item difficulty parameters (including their respective categories) and individuals’ abilities is iterative, by examining the relation with the probability of obtaining a specific score according to the individual’s ability. With this procedure, it is possible to obtain a value that better explains the achievement pattern registered. Simultaneously, it is possible to obtain the ability value for each individual according to the item difficulty pattern. This process was repeated by using the estimations of ability and difficulty until the iteration converged.

In the Rasch analysis, two basic fit statistics are employed: infit and outfit. These are calculated based on room mean squares, depending on the statistical value of Pearson’s chi-squared divided by the degrees of freedom, thus forming a scale with values ranging from 0 to infinity. Values below 1 indicate a higher than expected fit of the model, while values greater than 1 indicate a poor fit. Linacre (2002) suggested that those with values higher than 2 imply a bad fit to the model, making the conclusion of a reliable analysis impossible. For this reason, the authors of the present study used this value as a formal cut-off, both in items and subjects which, according to previous research, are also within the construct comparability approach (He et al., 2018). Moreover, the mean of individuals’ ability was set to 0 for the different subjects, as to allow the comparison of parameters estimations.

## Results

Before the implementation of the Rasch analysis, descriptive statistics of all subjects and participants for each province were observed. As seen in Table 1, the mean values are mostly located between 6 and 7.9, which is considered positive in terms of certification aptitude. Some exceptions are Geography and Greek Language, both from the humanities field, with 5.6 and 5.4, respectively.

**TABLE 1:** Examination grades by province: means and standard deviations.

	Alicante		Valencia		Castellón		Total participants	Total mean
Subject	Participants	Mean (SD)	Participants	Mean (SD)	Participants	Mean (SD)		
German language	1	7 (−)	8	7.63 (1.57)	1	9.3 (−)	10	7.74
Scenic arts	53	7.38 (1.47)	87	7.13 (1.34)	18	6.95	158	7.19
Biology	1745	6.19 (2.18)	2652	6.43 (2.15)	526	5.77 (2.34)	4923	6.28
Spanish language	6123	6.42 (1.65)	9510	6.14 (1.68)	2059	6.34 (1.66)	17692	6.26
Audiovisual culture	217	7.12 (1.56)	388	6.95 (1.67)	43	6.99 (1.26)	648	7.01
Design	187	7.08 (1.43)	328	6.27 (1.55)	45	6.59 (1.15)	560	6.56
Technical drawing	697	6.18 (2.34)	1348	6.50 (2.41)	218	6.83 (2.14)	2263	6.32
Economy	1698	6.47 (2.05)	3012	6.69 (1.91)	617	6.85 (1.87)	5327	6.64
Fundamentals of arts	249	6.78 (2.3)	392	6.78 (2.09)	70	6.11 (2.04)	711	6.71
Physics	1505	6.31 (2.41)	2312	6.35 (2.42)	457	6.43 (2.26)	4274	6.35
French language	161	7.43 (1.51)	231	8.14 (1.30)	73	8.42 (1.17)	465	7.94
Geology	94	5.8 (1.85)	69	5.18 (2.15)	14	6.33 (2.19)	177	5.6
Geography	1622	5.15 (2.41)	2882	5.57 (2.42)	600	5.02 (2.36)	5104	5.4
Greek language	539	6.62 (2.48)	730	6.39 (2.21)	155	6.67 (1.94)	1424	6.5
History of arts	715	5.79 (2.42)	926	6.10 (2.21)	181	6.35 (2.14)	1822	6
History of Spain	6124	7.03 (1.68)	9515	6.89 (1.66)	2060	6.97 (1.47)	17699	6.95
History of philosophy	780	6.38 (2.30)	1155	6.38 (2.16)	226	6.75 (2.33)	2161	6.42
English language	5960	6.71 (1.97)	9267	6.76 (1.92)	1986	6.86 (1.89)	17213	6.76
Italian language	4	7.4 (1.24)	4	9.06 (0.89)	0	−	8	8.23
Latin language	864	6.42 (2.18)	1098	6.31 (2.08)	249	6.91 (1.89)	1611	6.42
Mathematics	3180	7.17 (2.12)	5036	7.21 (2.16)	1074	7.40 (2.09)	9290	7.22
Applied mathematics	3180	5.47 (2.28)	3833	5.77 (2.25)	846	5.92 (2.06)	7859	5.69
to social sciences								
Chemistry	2182	5.70 (2.30)	3383	5.5 (2.31)	675	6.12 (2.22)	6240	5.77
Valencian language	4747	6.47 (1.54)	9048	6.38 (1.52)	1964	6.42 (1.63)	15759	6.42

*N, number of participants; M, mean; SD, standard deviation.*

It may also be noted that the number of participants presented an imbalance, due to the fact that students have to choose specific examinations. For example, the majority of students chose English Language, as it is mandatory for all educational centers. However, other languages, such as German or Italian, are not mandatory and are offered only by a few educational centers.

As the number of participants may affect the calculation parameter accuracy, there was a final selection that included those subjects with at least 1500 participants. For the Rasch PCM analysis, the subjects of German Language, Scenic Arts, Audiovisual Culture, Design, Fundamentals of Arts, French Language, Geology, Greek Language, and Italian Language were removed to improve accuracy.

The Rasch PCM analysis showed the summary statistics, including person reliability and separation indexes of 0.74 and 1.69, respectively. These values can be considered low, which indicates that the group of subjects was not sensitive enough to appropriately distinguish students with high and low achievement (Bond and Fox, 2007).

With respect to the unidimensionality of the model based on principal component analysis of residual scores, the results show a principal factor that explains 51.3% of the variance of the latent trait. With respect to a hypothetical second factor, it shows a value lower than 2 (Eigenvalue V2 = 1.4), which confirms the unidimensionality of the model.

In Table 2, examinations are ordered by their difficulty parameter (from high to low), together with their respective fit indexes. An optimal fit can be observed according to the established criteria. The examinations with a higher difficulty level were Chemistry, Geography, and Physics, whereas those with a lower difficulty level were Mathematics, History of Spain, and Economy.

**TABLE 2:** Difficulty parameters and fit statistics of the University Entrance Examinations.

Examinations	Difficulty	Infit	Outfit
Chemistry	−0.09	0.94	0.93
Geography	−0.23	1.43	1.42
Physics	−0.30	1.22	1.21
Applied mathematics to social sciences	−0.33	1.25	1.24
Biology	−0.35	0.92	0.90
Technical drawing	−0.41	1.58	1.56
Spanish language	−0.45	0.70	0.72
Art history	−0.47	1.24	1.23
Valencian language	−0.52	0.70	0.72
Latin language	−0.60	1.16	1.14
History of philosophy	−0.64	1.29	1.25
English language	−0.68	1.15	1.14
Economy	−0.73	0.98	0.97
History of Spain	−0.78	0.89	0.91
Mathematics	−0.83	1.28	1.22

Within the PCM framework, Table 3 shows the average of the category parameters that are used to estimate fit statistics, showing nearly perfect infit and outfit values. Moreover, the observed average of the measures – a description of the sample expected to increase with category value, as in this case – is computed and modeled to produce the responses observed in the category. Andrich Thresholds (also called step difficulty, step calibration or Rasch-Andrich threshold) are based on the calibrated measure of the transition from one category below to another adjacent category – the point on the latent variable at which adjacent categories are equally probable to be observed. For this reason, it indicates the difficulty to observe a specific category and not the difficulty to respond to this category (Linacre, 2019, p. 532). Step calibrations show that category ordering is interrupted only in the pair categories 3 and 4 (−0.86). However, this value is strictly influenced by the distribution of frequencies of observations in each category. As the average measures of the persons advance across categories, it can be assumed that the categories support monotonicity (Linacre, 2019, p. 532).

**TABLE 3:** Summary of category structure.

Score	Observed	Observed	Infit	Outfit	Andrich	Category
	count	average			threshold	measure
0	411	–0.54	1.35	1.37	–	–3.14
1	1037	–0.50	1.02	1.04	–1.62	–1.90
2	3143	–0.29	1.01	1.01	–1.49	–1.25
3	4889	–0.14	0.98	0.99	–0.65	–0.83
4	10994	0.03	0.99	0.99	–0.86	–0.46
5	15014	0.19	0.93	0.91	–0.19	–0.10
6	23062	0.40	0.97	0.96	–0.13	0.29
7	19242	0.63	0.96	0.96	0.69	0.73
8	22196	0.91	0.97	0.97	0.61	1.28
9	12055	1.22	1.02	1.01	1.66	2.07
10	6855	1.57	1.10	1.06	1.98	3.44

**FIGURE 1:**
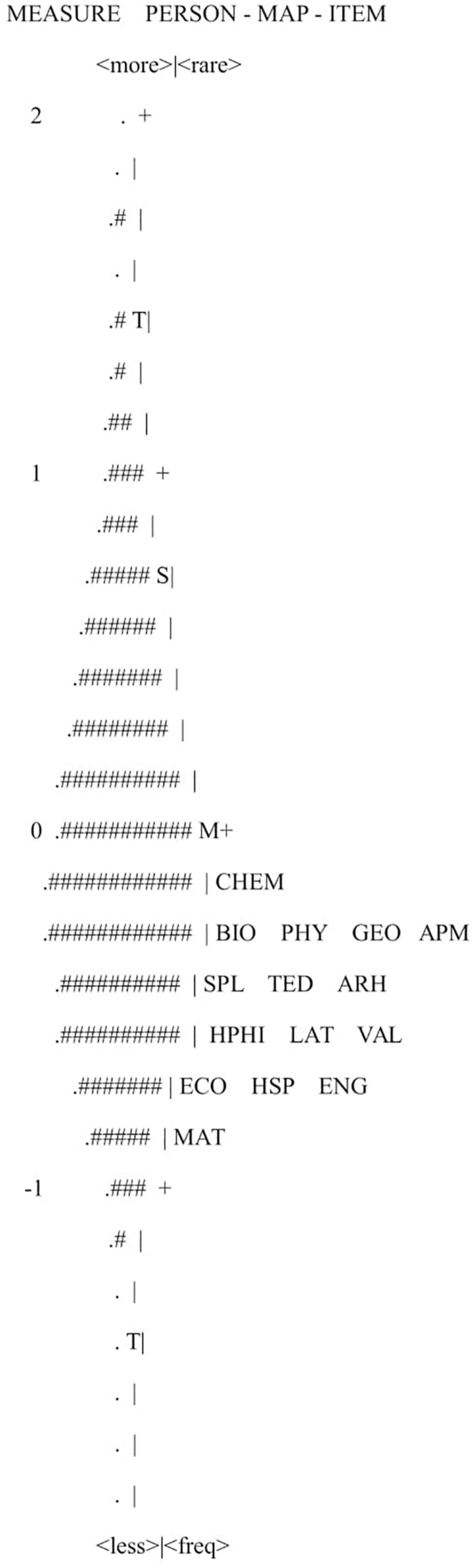
Item-Person map. EACH “#” IS 146: EACH “.” IS 1 TO 145. CHEM, chemistry; BIO, biology; PHY, physics; GEO, geography; APM, applied mathematics to social sciences; SPL, spanish language; TED, technical drawing; ARH, art history; HF, history of philosophy; LAT, lating language; VAL, valencian language; ECO, economy; HSP, history of Spain; ENG, english language; MAT, mathematics.

Figure 1 shows the “Wright map,” where persons and items are distributed along the ability and difficulty range, respectively. Persons are located on the left side of the graph, whereas examinations are located on the right side. It is noted that the difficulty of the examinations corresponded to persons’ abilities between log its 0 and −1. This may be positive, as most persons had sufficient ability to take the examinations. However, it also means that these examinations cannot accurately differentiate persons located at the top of the ability continuum.

## Discussion

This study aimed to analyze an empirical estimation of the qualifications obtained in the Spanish University Entrance Examinations with the application of the Rasch PCM, following the theoretical framework of the construct comparability approach. It is assumed that the measurement of academic achievement is a latent construct, allowing the comparison of difficulty parameters obtained for each of the standard scores for the corresponding subjects. This model has been considered useful in the assessment field for access to higher education (Tognolini and Andrich, 1996). The measurement system produced has been employed in different certificate examinations in many countries, including Tasmania (Tasmanian Qualification Authority [TQA], 2007) and England (Coe, 2008).

Following the first objective, the analyses showed the accomplishment of criteria of unidimensionality, which is essential for the application of the Rasch model, and the possibility of using a defined latent construct, namely academic achievement, in the PAU context. However, it must be noted that the establishment of this operative construct cannot be interpreted as the existence of a unique global process. The scientific literature posits that the interpretation of this construct is not clear, as it does not serve as the basis for the specific purpose of each examination (He et al., 2018). For this reason, it is argued that, although all examinations require specific abilities, they also demand global cognitive processes related to the construct measurement.

With respect to the second objective, an optimal fit was observed in all the examinations, which led to considering the invariance properties assumed by the Rasch model in terms of person and item (or examination) comparison along the same latent construct (Bond and Fox, 2007). Therefore, the consequences of this type of estimation lie in the possibility of making inferences beyond the students’ sample employed. At the same time, the examination fit allowed a comparison among them in terms of the difficulty parameters obtained together with the ability levels required to attain each possible score. From these results, a key concept was formed in the context of PAU – the selection of examinations, a topic widely discussed in international literature (Lamprianou, 2009). Bell et al. (2007) indicated that the perceived difficulty of a student in one or various examinations could be an obstacle to university entrance; as a consequence, other subjects might be favored with a higher enrollment fee. Taking into account the results of the present study, this may be happening with the subject of History of Spain to the detriment of History of Philosophy, as the students have to choose one and the number of candidates in the former is three times higher than the latter.

The analysis of the third objective highlights the need to consider the qualification scale employed in PAU as typical. Disorder rating category is observed between grades 3 and 4, which means that the 10-point category does not discriminate in some points of the latent trait. However, fit values were good for all categories, and the observed average of the measures increased with category values. It must be mentioned that the majority of countries that use comparative analysis employ a minor number of qualification categories. In this case, a smaller sample size may interfere with Andrich Threshold estimations. For this reason, in order to make similar estimations, future studies should analyze the general category structure in all Spanish communities that conclude general psychometric strategies.

Finally, the person separation index is low, showing that these examinations do not accurately differentiate students with high and low achievement. However, the Wright Map indicated that the difficulty levels of all examinations are within the students’ ability range; therefore, there are adequate probability levels of obtaining positive results. The location of the examinations on the scale corresponds to a similar distribution of the categories on the latent construct. Again, these results showed the need to recodify the category system to improve the differentiation of individual levels, as a higher number of students might be included for each of the high and low categories.

## Conclusion

In conclusion, the intention of this study was to initiate an effective analysis of standard scores comparison in Spain under the construct comparability approach, a theoretical and methodological framework used in other countries (Newton, 2012). Limitations and future directions should be addressed. First, the samples utilized in other countries are considerably larger, as data were collected throughout the country, which provides better estimations. This study is implemented within a single Spanish community, and it confirms the potential need for future studies similar to those conducted in England. In the Spanish context, it would be essential to draw a comparison between autonomous communities in order to find the appropriate equity measurement. This possibility has not been explored in the scientific literature in this field. However, considering that the majority of Spanish examinations have a written format, the differences between examiners in the interpretation of tasks and the evaluation categories by different raters, together with other possible effects (halo effect, gender, and cultural bias), may contribute to error measurement, validity and justice in evaluation (Frederiksen, 1984; Eckes, 2015). In this context, a multi-faceted Rasch model may adequately address these issues in the future.

## Data Availability Statement

The datasets for this manuscript are not publicly available because they belong to the Valencian Government who are restrictive to their data policy. Requests to access the datasets should be directed to University Regulation Service (sru@gva.es).

## Ethics Statement

The studies involving human participants were reviewed and approved by the University of Alicante. Written informed consent from the participants’ legal guardian/next of kin was not required to participate in this study in accordance with the national legislation and the institutional requirements.

## Author Contributions

AV: literature review, data analysis, statistical procedures, and manuscript writing. LN: literature review, manuscript writing, and manuscript revision. TP-R: manuscript writing and manuscript revision. PM: manuscript revision.

## Conflict of Interest

The authors declare that the research was conducted in the absence of any commercial or financial relationships that could be construed as a potential conflict of interest.
